# Soil seal development under simulated rainfall: Structural, physical and hydrological dynamics

**DOI:** 10.1016/j.jhydrol.2017.10.073

**Published:** 2018-01

**Authors:** Elena Armenise, Robert W. Simmons, Sujung Ahn, Amin Garbout, Stefan H. Doerr, Sacha J. Mooney, Craig J. Sturrock, Karl Ritz

**Affiliations:** a School of Energy, Environment, and Agrifood, Cranfield University, Bedford, UK; b Department of Geography, College of Science, Swansea University, Singleton Park, UK; c School of Biosciences, University of Nottingham, Sutton Bonington, Nottingham, UK

**Keywords:** Soil structural seal, Seal/crust thickness quantification, X-ray Computed Tomography (CT), Unsaturated hydraulic conductivity (K_un_), Soil water repellency, Simulated rainfall

## Abstract

This study delivers new insights into rainfall-induced seal formation through a novel approach in the use of X-ray Computed Tomography (CT). Up to now seal and crust thickness have been directly quantified mainly through visual examination of sealed/crusted surfaces, and there has been no quantitative method to estimate this important property. X-ray CT images were quantitatively analysed to derive formal measures of seal and crust thickness. A factorial experiment was established in the laboratory using open-topped microcosms packed with soil. The factors investigated were soil type (three soils: silty clay loam – ZCL, sandy silt loam – SZL, sandy loam – SL) and rainfall duration (2–14 min). Surface seal formation was induced by applying artificial rainfall events, characterised by variable duration, but constant kinetic energy, intensity, and raindrop size distribution. Soil porosities derived from CT scans were used to quantify the thickness of the rainfall-induced surface seals and reveal temporal seal micro-morphological variations with increasing rainfall duration. In addition, the water repellency and infiltration dynamics of the developing seals were investigated by measuring water drop penetration time (WDPT) and unsaturated hydraulic conductivity (K_un_). The range of seal thicknesses detected varied from 0.6 to 5.4 mm. Soil textural characteristics and OM content played a central role in the development of rainfall-induced seals, with coarser soil particles and lower OM content resulting in thicker seals. Two different trends in soil porosity vs. depth were identified: *i*) for SL soil porosity was lowest at the immediate soil surface, it then increased constantly with depth till the median porosity of undisturbed soil was equalled; *ii*) for ZCL and SL the highest reduction in porosity, as compared to the median porosity of undisturbed soil, was observed in a well-defined zone of maximum porosity reduction c. 0.24–0.48 mm below the soil surface. This contrasting behaviour was related to different dynamics and processes of seal formation which depended on the soil properties. The impact of rainfall-induced surface sealing on the hydrological behaviour of soil (as represented by WDTP and K_un_) was rapid and substantial: an average 60% reduction in K_un_ occurred for all soils between 2 and 9 min rainfall, and water repellent surfaces were identified for SZL and ZCL. This highlights that the condition of the immediate surface of agricultural soils involving rainfall-induced structural seals has a strong impact in the overall ability of soil to function as water reservoir.

## Highlights

• X-ray CT was effectively used to quantify soil seal/crust thickness.
• Different micro-morphological zones within seal layers were revealed.
• Rainfall had a strong and rapid impact on water transport and retention in soil.
• The existence of a soil-dependent raindrop impact threshold was hypothesized.

## 1 Introduction

A soil crust is a thin layer of consolidated material at the immediate soil surface with significantly different structural and mechanical characteristics than the underlying zone, which develops as result of temporal and spatial interactions between physical, biological and chemical properties and processes (Assouline, 2004, Bajracharya and Lal, 1999, Neave and Rayburg, 2007). In general, two broad classes of soil crusts are distinguished based on their predominant mechanism of formation, *viz.* biological and physical.

Biological soil crusts develop from the intimate association between soil particles and microorganisms such as cyanobacteria, green algae, fungi, bacteria, lichens and bryophytes, which live within or immediately on top of the uppermost millimetres of soil (Belnap and Gardner, 1993). They are typical of arid and semi-arid regions but can occur in most ecosystems (Bowker et al., 2010, Jeffery et al., 2009, Knapen et al., 2007). In contrast, physical crusts have a physicochemical origin (Neave and Rayburg, 2007). Rainfall impacting on the soil surface is one of the major drivers for physical seal formation. The kinetic energy associated with raindrop impacts destroys soil aggregates with concomitant compaction, slaking, physico-chemical dispersion and particle re-organization. Porosity at the immediate soil surface is significantly reduced through the in-filling and clogging of pores due to the wash-in of fine material, compaction associated with raindrop impacts, and deposition of clay particles at the immediate soil surface post-rainfall (Assouline, 2004). This gives rise to a *structural seal*. Dehydration of this rainfall-induced seal results in the formation of a *structural crust*. Throughout this paper the term seal is used when referring to the wetting phase, and crust when reference is made to the dehydrated seal.

The soil water reservoir is a major component of water storage within a catchment. Any alteration of the hydraulic structure of soils has great impacts on evapotranspiration, soil moisture content, groundwater recharge, runoff processes and river flow, and in turn largely influences the catchment water balance. Soil crusts, either biological or physical, are extremely frequent both on cultivated (Bajracharya and Lal, 1999, Bedaiwy, 2008, Knapen et al., 2007) and uncultivated soils (Menon et al., 2011). Therefore, the response of the soil surface to rainfall can have a significant impact on hydrological and ecological processes (Assouline et al., 2015). Recent record-breaking weather phenomena and flooding events in the UK and across Europe (Slingo, et al., 2014, Schneider et al., 2013) have renewed interest on the possible links between agricultural land management and flooding. In this context, the effects of biological and/or physical sealing on agricultural soils and its implications for downstream flood risk could be of major significance.

Hydrological models are regularly used for flood forecasting. At the basis of hydrological modelling is the rainfall-runoff relationship of the catchment. This is a highly complex and non-linear hydrological phenomenon (Chen and Adams, 2006, Modarres and Ouarda, 2013) that describes the transformation of precipitation into discharge (Wagener and Wheater, 2004). The presence of a biological and/or physical seal/crust on the soil surface modifies the partitioning between infiltration and runoff and adds extra complexity to the system. Chen et al. (2013) applied a rainfall-runoff model to analyse the respective role of several factors, including the presence of a physical seal layer, on the hydrological response of a semiarid hillslope. They found that the seal layer controlled runoff generation to such an extent that runoff was not generated when the seal was excluded from the simulation. Assouline and Mualem (2006) investigated the combined effect of soil heterogeneity and surface seal formation on the rainfall-runoff relationship of a small hypothetical bare catchment and showed that soil sealing had a bigger impact on runoff than soil heterogeneity.

The thickness of the disturbed layer is a key feature of a sealed surface, which allows estimation of the extent of the impact of the sealing phenomenon. Wide ranges in seal thickness have been reported in the literature. Visual examination and measurement of the sealed/crusted surface, either directly with Vernier calipers (Bedaiwy, 2008, Roth, 1997), or via microphotographs (Bresson and Boiffin, 1990, Bu et al., 2013), have been primarily used to directly quantify seal and crust thickness. However, the risk with any visual assessment method is an underestimation of the thickness of the disturbed layer, whilst the unobserved remaining disturbed part can still affect the flow processes in the upper soil layer. Bresson et al. (2004) showed the potential of X-ray techniques to characterise the bulk densit, of structural crusts. Here we describe the development of a method for a formally prescribed quantification of soil seal thickness which uses X-ray Computed Tomography (CT) data in an innovative way and enables a non-subjective assessment of seal formation and consolidation.

One of the principal constraints when describing and characterising sealed soils is the temporal and spatial variability of physical seal hydraulic properties and associated seal structure (Augeard et al., 2007). To overcome this limitation, Assouline and Mualem, 1997, Assouline and Mualem, 2000 developed and tested a model for soil sealing that takes into account several soil- and rainfall-related factors involved in seal formation. This model provided a much-needed theoretical basis for interpreting results from infiltration experiments in sealed soils and was successfully applied to simulate flow processes under sealing conditions. Small-scale experiments with simulated rainfall have shown that relatively stable seals might form in a relatively short time (Bu et al., 2013, Neave and Rayburg, 2007). Nevertheless, beyond this time seal development might continue, and a dynamic balance between seal destruction and formation might be established. With regards to spatial variability, it is likely that gradual changes of structure within the seal are more likely to occur rather than the two discrete-layer structure firstly described by McIntyre, 1958a, McIntyre, 1958b. Over the last few decades several studies have been conducted to investigate the processes and factors involved in seal formation (see Assouline, 2004). However, experiments that provide additional evidence of the micro-scale modifications of soil surface structure and wider implications for soil hydrodynamics at the initial stages of seal formation are still required. Accordingly, we conducted a laboratory experiment and induced surface seal development in three agricultural soils of contrasting texture with controlled rainfall events of constant intensity and kinetic energy (KE) and short storm duration time increments. Micro-morphological zones within the seal were identified non-destructively using X-ray CT and the micro-morphological characteristics of the seal quantified. Hydrological characterisation of the developing seals was conducted by measuring water repellency, a common feature of many soils (Doerr et al., 2000) and previously observed on soil crusts (Fischer et al., 2010), and by quantifying water infiltration dynamics.

## 2 Materials and methods

### 2.1 Soil sampling and preparation

Three soils of contrasting chemical and physical properties (a silty clay loam, sandy silt loam and sandy loam, denoted ZCL, SZL, SL hereafter; Table 1) were sampled from fields used in intensive horticultural production and known to be susceptible to surface sealing. The fields were situated at three different locations in the UK. The ZCL and SZL were obtained from Butterwick (52° 59′ 12″ N, 0° 3′ 33″ E) and Wragg Marsh Spalding (52° 49′ 50″ N, 0° 5′ 32″ W), Lincolnshire and classified as Wisbech series (Soil Survey of England and Wales) and Wisbech/Agney series respectively. SL was classified as Eardiston series, and sampled from Coughton, Ross-on-Wye, Herefordshire (51° 53′ 43″ N, 2° 33′ 50″ W). For each soil, a randomly selected 5-point bulk composite sample (60 kg) was collected (0–30 cm depth). Soil was stored overnight at 10 °C, and then sieved moist to <2 mm. Sieved soil was stored at 4 °C until required.

**Table 1:** Selected soils chemical and physical characteristics.

	ZCL	SZL	SL
*Particle size distribution*			
% Sand	16	35.1	69.9
% Silt	62	52.9	18.1
% Clay	22	12	12
% Coarse sand (600–2000 µm)	0.2	0.1	4.5
% Medium sand (212–600 µm)	0.4	0.3	39.4
% Fine sand (63–212 µm)	15.5	34.7	26
Textural class (Soil Survey of England and Wales, UK)	Silty clay loam	Sandy silt loam	Sandy loam

*Chemical properties*			
OM (%)	2.24	2.4	1.46
CaCO_3_ (%)	2.16	1.89	0.43
TN (%)	0.150	0.168	0.102

Open-topped microcosms were constructed using 50 mm lengths of 46 mm internal diameter PVC pipe. A nylon mesh (1 mm aperture) was glued to the bottom of the microcosm. Each microcosm was packed with fresh soil at a bulk density of 1.2 g cm^−3^. A factorial experimental design was implemented using the three soil types, subjected to four rainfall durations up to 14 min maximum (see below) and three types of analysis (unsaturated hydraulic conductivity, X-ray CT and water repellency), each applied to three independent replicates (total n = 108).

### 2.2 Simulated rainfall application

A rainfall tower was used to generate artificial rainfall which reproduces the physical characteristics of natural rainfall under controlled laboratory conditions in relation to drop-size distribution, kinetic energy (KE) and intensity. The rainfall simulator consisted of a 0.5 m^2^ bed of hypodermic needles (BD Microlance 3, 21 g, 0.8 × 40 mm) arranged in an offset 2.0 × 2.5 cm grid, sited at 8.8 m elevation above the plane of impact (Withers et al., 2007). A stainless steel 4.0 mm aperture mesh was located 1 m below the needle bed to break up the rain droplets to produce a randomly-distributed range of droplet sizes. A constant head of 15 mm reverse osmosis (RO) treated water above the needle drain points was created in the needle bed, and the spatial arrangement of water-releasing hypodermic needles changed until a uniform and replicable rainfall intensity, drop size distribution and KE was achieved.

Drop size distribution and drop fall velocities were quantified with a laser optical disdrometer (LOD, model OTT-Parsivel 2, OTT Messtechnik, Kempten, Germany). The LOD was positioned at five locations within the experimental area (front, middle, back, left and right). For each point four measurements, of 60 s duration were taken.

The temporal development of structural seals was induced by applying a controlled rainfall event of intensity 60 mm h^−1^ and KE of 18.4 J m^−2^ mm^−1^ for either 2, 5, 9, or 14 min duration (hereafter denoted D2, D5, D9, D14, were D abbreviates ‘duration’). This frequency of measurement was chosen to characterise the most dynamic stages of seal formation, namely aggregate breakdown and particle re-organization. Drop size ranged from 0.063 to 3.75 mm with a median drop size (D_50_) of 2.04 mm.

### 2.3 Unsaturated hydraulic conductivity analysis

Philip, 1957, Philip, 1969 presented the first analytical solution to Richards’ equation for vertical and horizontal infiltration. This has an infinite series solution for cumulative infiltration that is a function of time. However, for simplification, the infinite series solution is approximated by retaining only the first two terms in the infinite series resulting in the following equation:

(1) $$I(t)=A_{0}t^{1/2}+\mathrm{Kt}$$

where *I*(*t*) is the cumulative infiltration at time t, *A*_0_ is the soil sorptivity, and *K* is the soil hydraulic conductivity.

An infiltration experiment using a mini disk infiltrometer (Decagon Devices, Washington, USA) was conducted in the laboratory. Soil cores were placed on a sand table set to a pressure head (*h*) of −0.03 m and the mini disk infiltrometer, adjusted to the same pressure head value, was used to supply water to the top of each core. Prior to analysis, the samples were kept on the sand table for 24 h in order to equilibrate their matric potential. A thin layer of sand (Garside 80EW, Leighton Buzzard, UK) was applied to the soil surface to increase contact with the disk of the mini-disk infiltrometer. The infiltrometer was carefully placed on the contact sand and held in place with a ring stand and clamp. The elapsed time and the water level in the reservoir of the mini disk infiltrometer were recorded at predetermined time intervals up to 2 h. Philip infiltration model was then fitted to the measured infiltration data using nonlinear parameter optimization in excel and the unsaturated K was derived.

Capillary theory can be used to estimate the size of pores excluded from the transmission of infiltrating water at differing pressure heads (Sauer and Logsdon, 2002). Assuming the pores are cylindrical, for a certain pressure head (h) the pore radii can be predicted from:

(2) $$r=-\frac{2\sigma \cos\alpha }{\rho \mathrm{gh}}$$

where *σ* is the surface tension of water (assumed to be 0.073 N m^−1^), *α* is the contact angle between water and the pore wall (assumed = 0°), *α* is the density of water (Mg m^−3^), and *g* is the gravitational acceleration (9.8 m s^−2^). From Equation 2, a pressure head of −0.03 m would exclude pores with diameters equal to or larger than 1.0 mm diameter. Luxmoore (1981) classified pore sizes of <10 µm as micropores, from 10 to 1000 µm as mesopores and > 1000 µm as macropores. According to this classification, measurements of unsaturated hydraulic conductivity at a pressure head of −0.03 m will give an estimate of the flow through meso- and micropores.

### 2.4 Assessment of soil water repellency

The soil cores were equilibrated to a constant temperature and relative humidity (20 ± 1 °C, RH 50 ± 10%) for 24 h and water drop penetration time (WDPT) tests (Doerr, 1998) were performed under these same conditions. The surface of each soil core was divided into six parts using a pie-shaped frame, of which three sections were used for WDPT test for the wet surface and another three for the dry surface. Three drops of distilled water (20 μl) were dispensed using a fixed-volume pipette (Multipette Plus, Eppendorf, Germany). Water was slowly dispensed to make a drop hang on the tip of the pipette, which was then placed carefully on the soil surface to avoid any forced-penetration by a high kinetic energy of the water drop. After a set of WDPT tests for wet surfaces, all samples were dried in a dry oven (25 ± 5 °C) for 48 h. Another set of WDPT tests for dry surfaces was conducted on the unused areas, using the same WDPT procedures.

### 2.5 X-ray Computed Tomography

Microcosms were scanned using a Phoenix Nanotom 180NF tomograph (GE Sensing & Inspection Technologies GmbH, Wunstorf, Germany). The field of view for each scan included the entire sample. The scanner consisted of a 180 kV nanofocus X-ray tube fitted with a diamond transmission target and a five megapixel flat panel detector (Hamamatsu Photonics KK, Shizuoka, Japan). A maximum X-ray energy of 130 kV and 100 µA was used to scan each soil core. A total of 1440 projection images were acquired over a 360° rotation. The resulting isotropic voxel edge length was 24 µm and total scan time was 47 min per core. Reconstruction of the projection images to produce three dimensional (3-D) volumetric data sets was performed using the software datos|rec (GE Sensing & Inspection Technologies GmbH, Wunstorf, Germany). The reconstructed CT volumes were visualized and quantified using ImageJ software (version 1.48 V) (Rasband, 2014). A volume of interest (VOI) of cubic shape (1300 pixels size per axis) was cropped in the CT scan image in the middle of the sample in order to exclude the sample frame.

### 2.6 Image processing, segmentation and analysis

Scanned gray-scale data of the VOI’s were segmented using the global thresholding method (Otsu’s algorithm) to separate solid and pore phases. The Otsu thresholding algorithm was provided in the ImageJ software (version 1.48 V) (Rasband, 2014). A morphological filter permitted detection of the soil surface and thus to define layers as sections parallel to this detected surface (Fohrer et al., 1999). Seventeen layers of 10 voxels each i.e. 240 µm thick were then produced. The detectable pore total volume (>0.014 mm pores) within each of those sections were measured using the BoneJ plugins (Doube et al., 2010). The porosity was calculated in each section by dividing the pore total volume by the volume of the section.

### 2.7 Seal thickness quantification: a new approach

**Fig. 1:**
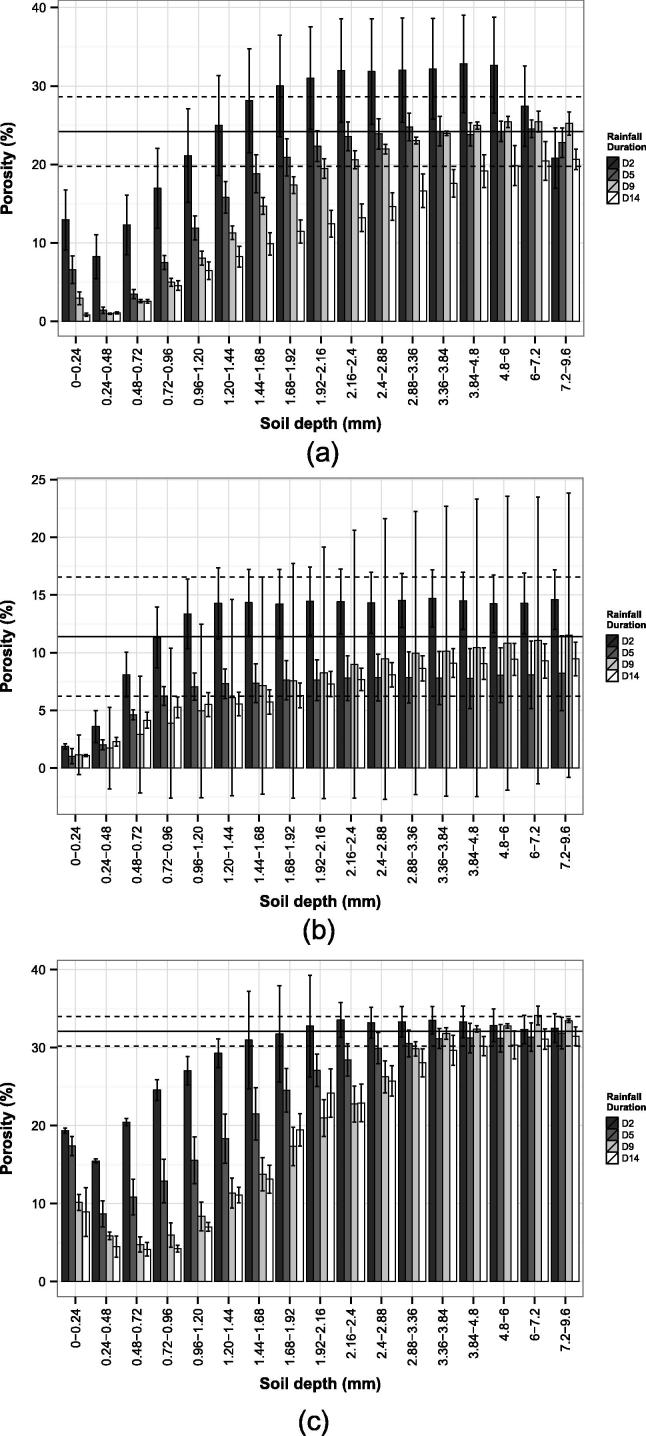
Variation of porosity of 17 soil layers in relation to simulated rainfall for 2 (D2), 5 (D5), 9 (D9) or 14 (D14) minutes duration. Bars denote median crust porosity (n = 3), whiskers denote ± confidence interval of the median. Horizontal solid line shows the median reference porosity ($\overset{\sim }{x}$φ*_ref_*), dashed lines show ± confidence interval around the $\overset{\sim }{x}$φ*_ref_*. (a) Silty clay loam (ZCL); (b) Sandy silt loam (SZL); (c) Sandy loam (SL).

Seal and crust thickness was derived using a method that employed values of porosity (φ) obtained from the CT data. A reference volume of soil (1 voxel), where by definition the disturbance by raindrop impact and the presence of washed-in particles from the surface were considered negligible, was identified in the lower part of the microcosms (36.3 mm below the soil surface) and its porosity calculated (reference porosity = φ*_ref_*). To identify seal thickness and micro-morphology, soil volumes were taken across the microcosm depth at increments of 24 µm. Consequently, 17 adjacent layers were considered in total and the corresponding porosities were calculated for each soil type and rainfall duration. These values of porosity were compared with the reference porosity (Fig. 1). For a specific soil and rainfall duration, the thickness of the seal corresponded to the depth at which the seal porosity returned to the median reference porosity ($\overset{\sim }{x}$φ*_ref_*) ± the median confidence interval (CI). For soils ZCL, SZL and SL the $\overset{\sim }{x}$φ*_ref_* ± CI values were 24% (±4.4%), 11% (±5%) and 32% (±1.9%), respectively (Fig. 1a–c).

### 2.8 Statistical analyses

One-way ANOVA was conducted to determine, for each soil type, the effects of rainfall duration on K_un_ and total porosity of the seal layer. The means were compared using Student–Newman–Keuls (SNK) test at the 0.05 level of significance. ANOVA assumptions of normality and homogeneity of variances of the residuals were explored graphically using respectively normal Q-Q plots and plots of the residuals versus fitted values. The Q-Q plot for K_un_ revealed log-normally distributed data therefore ANOVA for this variable was performed on log-transformed data. All statistical analyses were executed using R (R Development Core Team, 2010).

## 3 Results

### 3.1 Quantifying seal-thickness of different soils exposed to various durations of simulated rainfall

Fig. 1 shows the porosity calculated for each soil and rainfall duration and in relation to the superimposed $\overset{\sim }{x}$φ*_ref_* ± CI of the undisturbed soil matrix, and provides an alternative way to define soil and crust thickness. For the ZCL soil (Fig. 1a), at rainfall durations D2, D5, D9, and D14 the $\overset{\sim }{x}$φ*_ref_* ± CI was attained at 0.96–1.20, 1.68–1.92, 2.16–2.4, 4.8–6.0 mm respectively which suggests that the average seal thickness equated to 1.08, 1.8, 2.28, and 5.4 mm respectively.

**Fig. 2:**
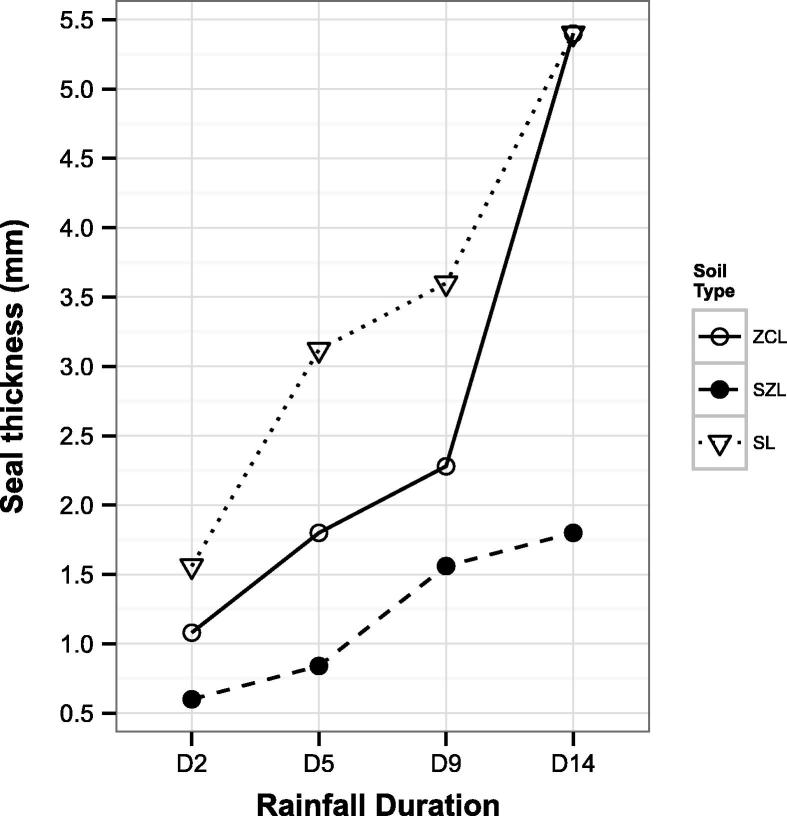
Seal thicknesses derived with the method proposed in this study and extrapolated from Fig. 1. One value of thickness for each soil type (Silty clay loam – ZCL; Sandy silt loam – SZL; Sandy loam – SL) and duration (2, 5, 9 and 14 min: D2, D5, D9, D14 respectively) of simulated rainfall.

Final average seal thickness values per rainfall duration and soil type derived using our imaging approach are represented in Fig. 2. For ZCL the estimated seal thickness increased with rainfall duration from 1.08 mm for rainfall duration D2 to 1.8 mm at D5, 2.28 mm at D9, and 5.4 mm at D14. Similarly, seal thickness in SL increased exponentially in the order D2 < D5 < D9 < D14 (1.56, 3.12, 3.6, 5.4 mm respectively). A different behaviour was observed for SZL, where the increment in seal thickness due to rainfall duration was very gradual (the seal developed was 0.6 mm thick at D2, 0.84 mm at D5, 1.56 mm at D9, and 1.8 mm at D14). The reduction in median porosity of the first 5 mm of soil with respect to the undisturbed zone was the lowest for SL (38%) and higher in ZCL (58%) and SZL (67%).

### 3.2 Spatial and temporal micro-morphological variations in seals due to rainfall

**Fig. 3:**
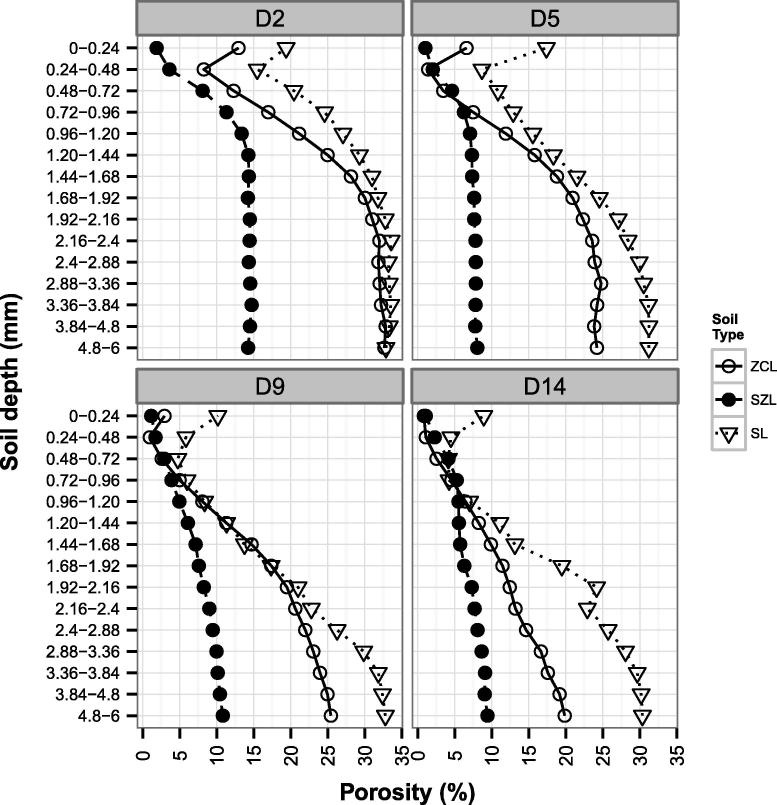
Porosity profiles with depth of the three soil types (Silty clay loam – ZCL; Sandy silt loam – SZL; Sandy loam – SL) subjected to different rainfall durations (2, 5, 9 and 14 min: D2, D5, D9, D14 respectively).

The dynamics of seal development and the predominant processes involved in rainfall-induced surface sealing can be inferred from Fig. 3. An abrupt and distinct zone of reduced porosity as compared with the $\overset{\sim }{x}$φ*_ref_*, located approximately 0.24–0.72 mm below the seal surface was identified for ZCL at D2, D5 and D9, and for SL at all durations.

After 2-min rainfall duration (D2) the immediate soil surface of SL (0.0–0.24 mm) was associated with a 40% reduction in porosity as compared with the median reference porosity ($\overset{\sim }{x}$φ*_ref_*). This trend continued throughout the rainfall event. Further, between 0.24 and 0.48 mm depth, porosity decreased to 16%, corresponding to a 52% reduction as compared with the $\overset{\sim }{x}$φ*_ref_*. This abrupt extra-dense zone was associated with a further 20% reduction in φ as compared with the soil surface. This zone of intense porosity reduction progressively increases achieving at D9 a maximum depth of 0.72–0.96 mm with % reductions in porosity relative to $\overset{\sim }{x}$φ*_ref_* ranging from 81 to 85% (Fig. 3). Below this zone of intense porosity reduction, an incremental increase in porosity was observed, with $\overset{\sim }{x}$φ*_ref_* being attained at 2.88–3.36 mm depth for D5, and 3.36–3.84 mm for D9 and D14. No significant differences in porosity values were observed between D9 and D14 for any of the depth intervals investigated (Fig. 1c). This suggested that optimum seal formation was attained following a 9 min storm duration.

As with the SL, for the ZCL the porosity of the uppermost soil layer progressively decreased with storm duration with porosity reductions as compared with $\overset{\sim }{x}$φ*_ref_* at D2, D5, D9 = D14 of 40%, 73% and 87%, respectively. The abrupt and narrow zone of intense porosity reduction was again observed between 0.24 and 0.48 mm depth at all storm durations achieving a maximum reduction in porosity as compared with $\overset{\sim }{x}$φ*_ref_* of >90% (Fig. 1a). In comparison with SL, no significant differences in porosity were observed between D9 and D14 for depth intervals 0.24–0.48 to 1.44–1.68 mm (Fig. 1a). However, in contrast to SL, further significant reductions in porosity were observed between D9 and D14 for all depth intervals between 1.68–1.92 and 4.80–6.00 mm. This inferred a continued in-washing of fines to depth.

SZL did not present the zone of intense porosity reduction below the soil surface, rather the highest porosity reduction was associated with the 0–0.24 mm layer. The surface reduction in φ for SZL as related to the $\overset{\sim }{x}$φ*_ref_* of 11% was equal to 84, 91, 90 and 91% at D2, D5, D9 and D14 respectively. In addition, at D5, D9 and D14 the porosity profiles with depth of SZL showed a very similar trend (Fig. 3), and similar porosities values were also observed for D5, D9 and D14 at all depth intervals (Fig. 1b). These results indicated that after 5 min additional rainfall did not significantly alter the sealing processes in SZL and the optimum seal formation occurred at D5.

### 3.3 Resultant effects of sealing upon infiltration and water repellency phenomena

**Fig. 4:**
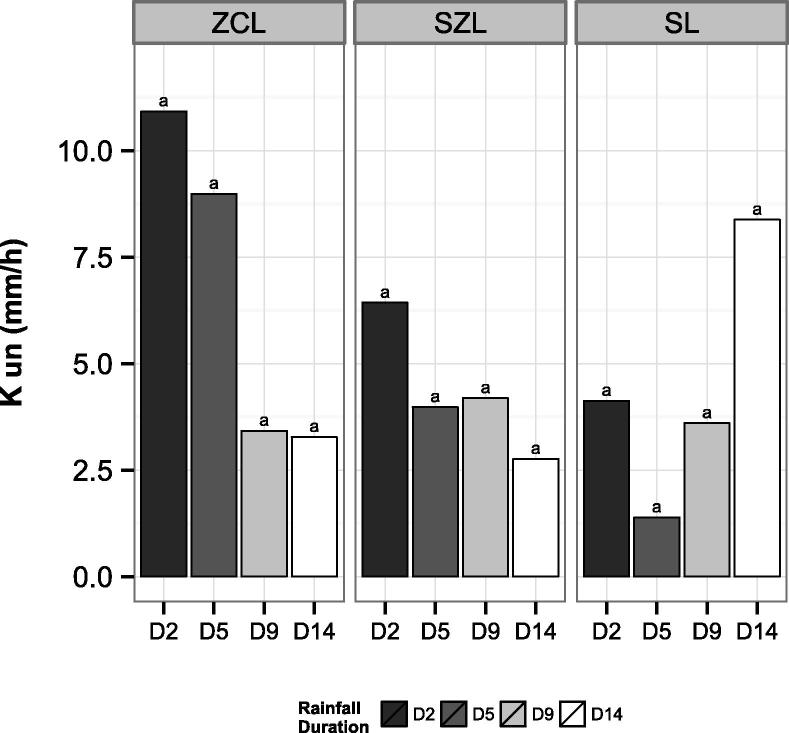
Effect of rainfall duration (2, 5, 9 and 14 min: D2, D5, D9, D14 respectively) and soil type (Silty clay loam – ZCL; Sandy silt loam – SZL; Sandy loam – SL) on the unsaturated hydraulic conductivity (K_un_) measured at −0.03 m pressure head. Different letters indicate differences at P < .05 according to SNK test performed on log-transformed data.

Generally, increasing rainfall duration appeared to reduce the unsaturated hydraulic conductivity (Fig. 4), although this trend was marginally statistically significant for ZCL and SZL (*P* = .10 and 0.09 respectively) and not significant for SL (*P* = .45). The highest reduction in hydraulic conductivity occurred between D5 and D9 for ZCL and between D2 and D5 for SZL: from 9.0 to 3.4 mm h^−1^ and 6.4 to 4.0 mm h^−1^ respectively. For SL, K varied between 1.4 and 8.4 mm h^−1^ showing no relevant trend. The lowest K_un_ was observed in SL (median of all durations equal to 2 mm h^−1^), followed by SZL (4.1 mm h^−1^) and ZCL (5.4 mm h^−1^).

**Fig. 5:**
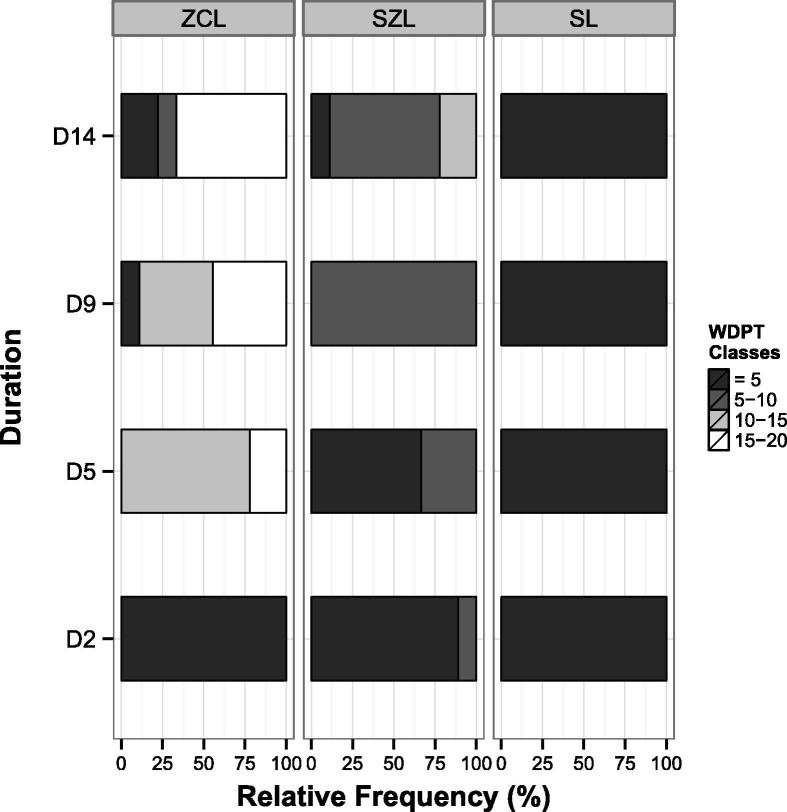
Relative frequency of four observed water-drop penetration time (WDPT) classes (<5 s, 5 s < WDPT <10 s, 10 s < WDPT <15 s, and 15 s < WDPT <20 s) for three soil types (Silty clay loam – ZCL; Sandy silt loam – SZL; Sandy loam – SL) at different rainfall durations (2, 5, 9 and 14 min: D2, D5, D9, D14 respectively).

Water drop penetration time (WDPT) data for wet surfaces only are shown in Fig. 5. Dry surfaces and those of untreated control cores showed no notable water repellency with most drops penetrating without measurable delay (WDPT range 0–6 s). Reduction or disappearance of water repellency at very low water contents has been previously documented (de Jonge et al., 1999). For each soil and rainfall duration, the relative frequencies of four penetration time classes were reported: WDPT < 5 s, 5 s < WDPT <10 s, 10 s < WDPT <15 s, and 15 s < WDPT <20 s. Drops lasting longer than 20 s were not observed for any soil surface. On average, the water repellency was greater in ZCL than SZL, with the greatest penetration times ranging between 15–20 s and 10–15 s respectively. SL showed no signs of water repellency at any rainfall durations (WDPT, <5 s). In general, with the exception of SL, increasing rainfall duration increased the proportion of more repellent classes, hence soil water repellency. In ZCL the relative frequency of the class 15–20 s increased from 0% at D2 to 22% at D5, 44% at D9, and 67% at D14. Similarly, in SZL the occurrence of penetration times between 5 and 10 s increased with duration as D2 > D5 > D9 (i.e. 11%, 33% and 100%) and decreased to 68% only at D14 where a longer time (10–15 s) appeared.

Based on the widely used WDPT classification system of Bisdom et al. (1993), all samples exhibited median penetration times classified either as wettable (WDPT < 5 s) or slightly water repellent (5–60 s). Water repellency clearly differed between the three soil types (Fig. 5), decreasing with the content of fine soil particle fractions (Table 1). Specifically, the content of silt and clay, equal to 84, 65, and 30% for ZCL, SZL and SL respectively, was positively related to the median WDPT measured for the three soils (13, 6 and 0 s).

## 4 Discussion

Crusts and seals should not be seen as discrete layers, but instead as disturbed layers that exhibit gradual changes in physical properties from the surface to some point beneath where the soil is still essentially undisturbed (Mualem et al., 1990). The ability to detect this potential pattern was at the core of the methodological approach based on CT-scan analysis for quantifying seal and crust thickness adopted in this study.

### 4.1 Seal thickness quantification and relationship with soil type and rainfall

The seal thickness measured with the method here developed ranged between 0.6 mm and 5.4 mm. Similarly to Bradford and Huang (1992), thick seals formed readily in the sandy loam soil (SL) which also had the lowest OM content. Comparatively thinner seals were observed in the sandy silt loam soil (SZL) and this may in part be due to the higher OM content that imparted higher aggregate stability; ZCL was mid-range in terms of seal thickness, and had an intermediate OM content. A wide range of seal thicknesses have been reported in the literature: between 1 mm and 8 mm (Bu et al., 2013), between 0.6 mm and 16.8 mm (Bedaiwy, 2008), 4 mm (Hyväluoma et al., 2012), between 0.2 mm and 20 mm (Bresson and Boiffin, 1990), between 2 mm and 12 mm (Roth, 1997). With the exclusion of Hyväluoma et al. (2012), all the aforementioned studies relied on visual examination for seal thickness quantification. The subjectivity associated with visual examination might have contributed to such variability in seal thickness reported in previous work, in conjunction with the obvious differences in soil type and experimental conditions used. Hyväluoma et al. (2012) used CT images to delineate the extent of the surface seal. They assessed the variation with depth of several soil properties, some directly determined from CT scans (porosity, pore size distribution), some derived after flow simulation through the pore structure obtained from the CT scans (permeability, tortuosity, effective porosity). They concluded that, given the good agreement of the various metrics used in estimating seal thickness (approximately 3 mm and 4 mm), these could be used for quantifying the thickness of a compacted layer. However, these authors acknowledged that the small number of samples used (four) limited the validity of the results.

Seal thickness was strongly influenced by the amount of rain impacting onto the soil surface, i.e. the higher the rainfall duration, the thicker the resultant seal. It is well established that the rate of seal expansion and thickening is controlled by rainfall characteristics. Farres (1978) observed soil crust thickness increased with the cumulative rainfall and suggested an empirical relationship for this phenomenon. Bedaiwy (2008) determined that the increase of seal thickness followed closely the increase in rainfall KE.

### 4.2 Seal micro-morphology and relationship with soil type and rainfall

Structural seals are formed at the soil surface by the destruction of soil aggregates exposed to the direct impact of rain drops, compaction, slaking, particle segregation, and pores filling and clogging by wash-in of fine material (McIntyre, 1958a, McIntyre, 1958b, Assouline, 2004). Badorreck et al. (2013) successfully used CT data to characterise and identify micro-morphological crust types of undisturbed soil samples from an artificial catchment. Similarly, in our study X-ray CT analysis was able to reveal the dynamics and processes of seal formation and differentiate micro-morphological zones within the seal. In two out of three soils under study, a zone of extreme porosity reduction was found at 0.24–0.72 mm below the soil surface. Our results corroborated with those of Fohrer et al. (1999), who derived the bulk density distribution with depth using a medical X-ray CT scanner and observed the maximum value of bulk density at 1 mm below the surface. This behaviour contrasted with previous results indicating that the maximum bulk density (and consequently lowest porosity) is reached at the soil surface itself. Fohrer et al. (1999) were not able to distinguish if the increased bulk density below the surface was either caused by a systematic image reconstruction error or by a real sealing pattern. This was because they used only one soil type and the phenomenon recurred in all the samples. In our study, SZL did not present the extra-dense layer below the surface. For this soil the porosity was lowest at the immediate surface and incrementally increased with depth. This indicated that the subsurface extra-dense layer detected by tomography was not a technical artefact, but a real micro-morphological soil property.

To understand the reason for the presence/absence of the zone of extreme porosity reduction below the soil surface, raindrop impact phenomena and the associated process of soil detachment should be taken into account. Erpul et al., 2008, Salles et al., 2000 explained that when a drop hits the soil surface, it penetrates the surface and compresses the soil. At the same time, during raindrop impact the pressure builds up at drop-soil interface and the high pressure inside the raindrop forces the water to escape laterally. The compressive stress is then transformed to shear stress across the solid-liquid contact region because of the lateral water movement. Basically, two stress components can be identified during raindrop impact: compressive stress from the impact (normal to the surface), shear stress from the lateral movement or jetting (tangential to the surface). The interaction between the obstacles (such as the bulge around the crater formed after drop hits and the irregularity of the soil surface itself) and the lateral jet stream is believed to be the major mechanism for soil detachment. Huang et al., 1982, Huang et al., 1983 showed that the partition of compressive and shear forces depends on the surface properties with greater lateral jet development, and in turn detachment, on a rigid surface compared to an elastic surface. More recently Erpul et al., 2003, Erpul et al., 2005) confirmed that the magnitude of compressive vs. shear stress components is dependent on the strength of the raindrop impacted surface and affects detachment.

It appears therefore that soil properties have a significant effect on the process of soil detachment as result of drop impact. Al-Durrah and Bradford (1982) reported that cohesion forces between soil particles strongly control the detached mass. When the disruptive forces from a raindrop overcome the bonding energy of the soil aggregates and the particle mass, soil detachment will take place (Lal, 1981). Evidence of a raindrop impact-threshold have been presented by several authors (Brandt, 1989, Sharma et al., 1991, Salles et al., 2000). In general, a sandy soil is less cohesive than a silt loam soil, and the minimum threshold rain energy needed to initiate soil detachment is lower (Salles et al., 2000).

It might be hypothesized that for the sandy silt loam soil (SZL) the critical KE required to initiate and support detachment was higher than the value used in our experiment (18.4 J m^−2^ mm^−1^), and thus soil detachment was minimal. Furthermore, due to specific textural characteristics, the predominant force acting at the immediate soil surface might have been the compressive component and this resulted in a general slumping of the soil core and produced a porosity vs. depth profile without zone of reduced porosity. On the other hand, ZCL and SL textural characteristics might have been associated with a lower raindrop impact threshold. At the same time, the lateral jet development on the surfaces of ZCL and SL might have been greater (hence a greater shear stress) which generated significant detachment. The raindrop compressive stress would have still been acting under the zone of influence of the lateral jetting, resulting in a rapid compaction/consolidation of soil. The loose soil particles at the immediate surface are expected to have a lower bulk density (and higher porosity) than the underling compressed zone, and this could be the reason for the abrupt and distinct zone of reduced porosity below the surface observed in the porosity vs. depth profiles of ZCL and SL.

Short rainfall durations (2, 5, 9 and 14 min) were selected to characterise the more dynamic stage of seal formation. It has already been established that relatively stable seals might form in a short time. Sang et al. (2008) showed the cumulative porosity of the 0–2 mm layer for soil either treated with anionic polyacrylamide (PAM) or untreated at four rainfall durations (7.5, 15, 30, 60 min). They reported the biggest drop in cumulative porosity between 7.5 and 15 min rainfall for both treated and untreated soil. In our study a 5 min rainfall was sufficient to induce the formation of 1.8, 0.84 and 3.12 mm seal in ZCL, SZL and SL soils respectively. The drastic reduction in porosity in the upper layers compared to the median porosity of undisturbed soil (median reference porosity = $\overset{\sim }{x}$φ*_ref_*) provided robust evidence of seal establishment. This was also supported by the steep decrement in water flow through meso- and micropores (indicated by unsaturated hydraulic conductivity measured at −0.03 m pressure head) between D2 and D9.

### 4.3 Infiltration dynamics and water repellency of developing seals

Rainfall had a rapid strong effect on the hydraulic properties of soil and this was clearly connected to the surface sealing process. After only 9 min, the unsaturated hydraulic conductivity was reduced by around 60% of its initial value. At this point in time the average seal thickness of the three soils was around 2.5 mm. Further seal expansion and modifications of the porous system due to the additional rainfall had a smaller impact on the K_un_.

Despite being a sandy loam soil and having the highest seal porosity, SL was associated with the lowest unsaturated hydraulic conductivity. This was attributed to the fact that K_un_ measurements at −0.03 m pressure head selectively excluded the water flow in pores ≥1 mm (i.e. the macropores). Given the textural characteristics of SL, it was likely that the relative abundance of macropores was higher than meso- and micropores. By excluding the macropores, the pore fraction contributing the most to the infiltration in SL was excluded, hence the low K_un_ recorded. This result stressed the importance of considering pores size when studying the hydrologic responses of surface seals. It also highlighted that a minimum of two pressure head points, characterising different pore size classes (e.g. 0.005–0.03 m), should be selected for a better representation of the unsaturated flow.

Soil water repellency reduces the affinity of a soil to water such that it resists wetting for periods ranging from a few seconds to hours, days or weeks. It can be caused by hydrophobic organic compounds present on soil particle surfaces or as interstitial material (Doerr et al., 2000). The importance of this reduced soil wettability on surface runoff generation has been widely highlighted (Cerda et al., 1998), including that of low levels of water repellency, which can also affect soil water distribution (Müller et al., 2007). Doerr et al. (2003) have thus called for the inclusion of this variable in hydrological modelling to improve model predictions in catchments with water repellent soils.

Although soil water repellency has often been thought to develop more readily in coarse-textured soils due to their smaller surface area per unit volume (Crockford et al., 1991, De Bano, 1981) our results support previous findings where water repellency increased in soil particle fractions of decreasing size (de Jonge et al., 1999, Doerr et al., 1996, Mataix-Solera and Doerr, 2004, Rodríguez-Alleres et al., 2007). Modifications of the soil particle size distribution, caused by the destruction of aggregates with raindrops impact, might be the cause of the development of water repellency following rainfall and its increase with rainfall duration. Rainfall might have preferentially exposed the finest and more hydrophobic fractions, as well as the interstitial organic matter, and in turn affected soil water repellency.

## 5 Conclusions

In this study we have developed and tested a new imaging approach for quantifying seal/crust thickness, which enabled us to characterise soil seal formation under simulated rainfall at a fine spatial and temporal scale.

The seals formed under the experimental conditions produced in the laboratory ranged between 0.6 and 5.4 mm in thickness and, in general, the increase of thickness followed the increase in rainfall duration. The sandy loam soil, which was also characterised by a very low OM content, developed thicker seals, whilst the sandy silt loam soil with the higher OM developed thinner seals.

By coupling short rainfall duration time increments with CT-scan technology it was possible to illustrate the structural seal formation process and the temporal inter-related dominance and significance of the associated sub-processes. Critically, in the ZCL and SL soils, we were able to identify a distinct and persistent zone of intense porosity reduction immediately below the seal surface (0.24–0.48 mm). This result confirmed the existence of a raindrop impact threshold (related to a critical KE) that needs to be overcome in order to initiate and support soil detachment and that is specific for each soil type. Rainfall duration had a rapid strong effect on the hydraulic properties and water repellency of soil: unsaturated hydraulic conductivity was reduced and WDPT increased as rainfall duration increased. These results were related to rainfall-induced aggregate breakdown processes and, in general, seal formation.

Structural sealing can occur even at very short rainfall durations. In this study <9 min rain was sufficient to induce the formation of an average a >2.5 mm thick seal in three different soils and cause a 60% reduction of infiltration. These results demonstrate how the surface condition of intensively cultivated soils can have a profound effect on the potential ability of soils to act as a water reservoir, with wider implications for flood-risk management in catchments.

## References

[b0005] Al-Durrah M.M., Bradford J.M. (1982). The mechanism of raindrop splash on soil surfaces. Soil Sci. Soc. Am. J..

[b0010] Assouline S. (2004). Rainfall-induced soil surface sealing: a critical review of observations, conceptual models, and solutions. Vadose Zone J..

[b0015] Assouline S., Mualem Y. (1997). Modeling the dynamics of seal formation and its effect on infiltration as related to soil and rainfall characteristics. Water Resour. Res..

[b0020] Assouline S., Mualem Y. (2000). Modeling the dynamics of seal formation: analysis of the effect of soil and rainfall properties. Water Resour. Res..

[b0025] Assouline S., Mualem Y. (2006). Runoff from heterogeneous small bare catchments during soil surface sealing. Water Resour. Res..

[b0030] Assouline S., Thompson S.E., Chen L., Svoray T., Sela S., Katul G.G. (2015). The dual role of soil crusts in desertification. J. Geophys. Res. Biogeosci..

[b0035] Augeard B., Assouline S., Fonty A., Kao C., Vauclin M. (2007). Estimating hydraulic properties of rainfall-induced soil surface seals from infiltration experiments and X-ray bulk density measurements. J. Hydrol..

[b9000] Badorreck A., Gerke H.H., Hüttl R.F. (2013). Morphology of physical soil crusts and infiltration patterns in an artificial catchment. Soil Till. Res..

[b0040] Bajracharya R.M., Lal R. (1999). Land use effects on soil crusting and hydraulic response of surface crusts on a tropical Alfisol. Hydrol. Process..

[b0045] Bedaiwy M.N.A. (2008). Mechanical and hydraulic resistance relations in crust-topped soils. Catena.

[b0050] Belnap J., Gardner J.S. (1993). Soil microstructure in soils of the Colorado plateau – the role of the cyanobacterium *Microcoleus vaginatus*. West. N. Am. Nat..

[b0055] Bisdom E.B.A., Dekker L.W., Schoute J.F.T. (1993). Water repellency of sieve fractions from sandy soils and relationships with organic material and soil structure. Geoderma.

[b0060] Bowker M.A., Maestre F.T., Escolar C. (2010). Biological crusts as a model system for examining the biodiversity–ecosystem function relationship in soils. Soil Biol. Biochem..

[b0065] Bradford J.M., Huang C. (1992). Mechanisms of crust formation: physical components. Soil Crust. Chem. Phys. Processes.

[b0070] Brandt C.J. (1989). The size distribution of throughfall drops under vegetation canopies. Catena.

[b0075] Bresson L.M., Boiffin J. (1990). Morphological characterization of soil crust development stages on an experimental field. Geoderma.

[b0080] Bresson L.M., Moran C.J., Assouline S. (2004). Use of bulk density profiles from x-radiography to examine structural crust models. Soil Sci. Soc. Am. J..

[b0085] Bu C.-F., Gale W.J., Cai Q.-G., Wu S.-F. (2013). Process and mechanism for the development of physical crusts in three typical Chinese soils. Pedosphere.

[b0090] Cerda A., Schnabel S., Ceballos A., Gomez-Amelia D. (1998). Soil hydrological response under simulated rainfall in the Dehesa land system (Extremadura, SW Spain) under drought conditions. Earth Surf. Proc. Land.

[b0095] Chen J., Adams B.J. (2006). Integration of artificial neural networks with conceptual models in rainfall-runoff modeling. J. Hydrol..

[b0100] Chen L., Sela S., Svoray T., Assouline S. (2013). The role of soil-surface sealing, microtopography, and vegetation patches in rainfall-runoff processes in semiarid areas. Water Resour. Res..

[b0105] Crockford H., Topalidis S., Richardson D.P. (1991). Water repellency in a dry sclerophyll eucalypt forest – measurements and processes. Hydrol. Process..

[b0110] De Bano, L.F., 1981. Water Repellent Soils: A State-of-the-Art. US Department of Agriculture, Forest Service, General Technical Report (PSW-46).

[b0115] de Jonge L.W., Jacobsen O.H., Moldrup P. (1999). Soil water repellency: effects of water content, temperature, and particle size. Soil Sci. Soc. Am. J..

[b9005] Doerr S.H. (1998). On standardising the “Water Drop PenetrationTime” and the “Molarity of an Ethanol Droplet” techniques to classify soil water repellency: a case study using mediumtextured soils. Earth Surf. Proc. Land.

[b0120] Doerr S.H. (2003). Soil water repellency as a potential parameter in rainfall-runoff modelling: experimental evidence at point to catchment scales from Portugal. Hydrol. Process..

[b0125] Doerr S.H., Shakesby R.A., Walsh R.P.D. (1996). Soil hydrophobicity variations with depth and particle size fraction in burned and unburned Eucalyptus globulus and Pinus pinaster forest terrain in the Agueda Basin, Portugal. Catena.

[b0130] Doerr S.H., Shakesby R.A., Walsh R.P.D. (2000). Soil water repellency: Its causes, characteristics and hydro-geomorphological significance. Earth Sci. Rev..

[b0135] Doube M., Klosowski M.M., Arganda-Carreras I., Cordelieres F.P., Dougherty R.P., Jackson J.S., Schmid B., Hutchinson J.R., Shefelbine S.J. (2010). BoneJ: free and extensible bone image analysis in ImageJ. Bone.

[b0140] Erpul G., Norton L.D., Gabriels D. (2003). The effect of wind on raindrop impact and rainsplash detachment. Trans. ASAE (Am. Soc. Agric. Eng.).

[b0145] Erpul G., Gabriels D., Norton L.D. (2005). Sand detachment by wind-driven raindrops. Earth Surf. Proc. Land..

[b0150] Erpul G., Gabriels D., Cornelis W.M., Samray H.N., Guzelordu T. (2008). Sand detachment under rains with varying angle of incidence. Catena.

[b0155] Farres P. (1978). Role of time and aggregate size in the crusting process. Earth Surf. Proc..

[b0160] Fischer T., Veste M., Wiehe W., Lange P. (2010). Water repellency and pore clogging at early successional stages of microbiotic crusts on inland dunes, Brandenburg, NE Germany. Catena.

[b0165] Fohrer N., Berkenhagen J., Hecker J.M., Rudolph A. (1999). Changing soil and surface conditions during rainfall single rainstorm/subsequent rainstorms. Catena.

[b0170] Huang C., Bradford J.M., Cushman J.H. (1982). A numerical study of raindrop impact phenomena: the rigid case. Soil Sci. Soc. Am. J..

[b0175] Huang C., Bradford J.M., Cushman J.H. (1983). A numerical study of raindrop impact phenomena: the elastic deformation case. Soil Sci. Soc. Am. J..

[b0180] Hyväluoma J. (2012). Using microtomography, image analysis and flow simulations to characterize soil surface seals. Comput. Geosci..

[b0185] Jeffery S., Harris J.A., Rickson R.J., Ritz K. (2009). The spectral quality of light influences the temporal development of the microbial phenotype at the arable soil surface. Soil Biol. Biochem..

[b0195] Knapen A., Poesen J., Galindo-Morales P., De Baets S., Pals A. (2007). Effects of microbiotic crusts under cropland in temperate environments on soil erodibility during concentrated flow. Earth Surf. Proc. Land..

[b0200] Lal R., Morgan R.P. (1981). Soil conservation: preventive and control measures. Soil Conservation Problems and Prospects.

[b0205] Luxmoore R.J. (1981). Micro, meso-, and macroporosity of soil. Soil Sci. Soc. Am. J..

[b0210] Mataix-Solera J., Doerr S.H. (2004). Hydrophobicity and aggregate stability in calcareous topsoils from fire-affected pine forests in southeastern Spain. Geoderma.

[b0215] McIntyre D.S. (1958). Permeability measurements of soil crusts formed by raindrop impact. Soil Sci..

[b0220] McIntyre D.S. (1958). Soil splash and the formation of surface crusts by raindrop impact. Soil Sci..

[b0225] Menon M. (2011). Assessment of physical and hydrological properties of biological soil crusts using X-ray microtomography and modeling. J. Hydrol..

[b0230] Modarres R., Ouarda T.B.M.J. (2013). Modeling rainfall-runoff relationship using multivariate GARCH model. J. Hydrol..

[b0235] Mualem Y., Assouline S., Rohdenburg H. (1990). Rainfall induced soil seal (C) A dynamic model with kinetic energy instead of cumulative rainfall as independent variable. Catena.

[b0240] Müller K., Carrick S., Meenken E., Clemens G., Hunter D., Rhodes P., Thomas S. (2016). Is subcritical water repellency an issue for efficient irrigation in arable soils?. Soil Tillage Res..

[b0245] Neave M., Rayburg S. (2007). A field investigation into the effects of progressive rainfall-induced soil seal and crust development on runoff and erosion rates: the impact of surface cover. Geomorphology.

[b0250] Philip J.R. (1957). The theory of infiltration: 4. Sorptivity and algebraic infiltration equations. Soil Sci..

[b0255] Philip J.R. (1969). Theory of infiltration. Adv. Hydrosci..

[b0260] R Development Core Team (2010). R: A Language and Environment for Statistical Computing.

[b0265] Rasband, W.S., 1997–2016. ImageJ. US National Institutes of Health, Bethesda, Maryland, US. Retrieved from: https://imagej.nih.gov/ij/.

[b0270] Rodríguez-Alleres M., de Blas E., Benito E. (2007). Estimation of soil water repellency of different particle size fractions in relation with carbon content by different methods. Sci. Total Environ..

[b0275] Roth C.H. (1997). Bulk density of surface crusts: depth functions and relationships to texture. Catena.

[b0280] Salles C., Poesen J., Covers G. (2000). Statistical and physical analysis of soil detachment by raindrop impact: rain erosivity indices and threshold energy. Water Resour. Res..

[b0285] Sang S.L., Gantzer C.J., Thompson A.L., Anderson S.H., Ketcham R.A. (2008). Using high-resolution computed tomography analysis to characterize soil-surface seals. Soil Sci. Soc. Am. J..

[b0290] Sauer T.J., Logsdon S.D. (2002). Hydraulic and physical properties of stony soils in a small watershed. Soil Sci. Soc. Am. J..

[b0295] Schneider C., Laize C.L.R., Acreman M.C., Florke M. (2013). How will climate change modify river flow regimes in Europe. Hydrol. Earth Syst. Sci..

[b0300] Sharma P.P., Gupta S.C., Rawls W.J. (1991). Soil detachment by single raindrops of varying kinetic energy. Soil Sci. Soc. Am. J..

[b0305] Slingo, J. et al., 2014. The recent storms and floods in the UK, Met Office, http://www.metoffice.gov.uk/research/news/2014/uk-storms-and-floods.

[b0310] Wagener T., Wheater H.S. (2004). Rainfall-Runoff Modelling in Gauged and Ungauged Catchments.

[b0315] Withers P.J.A., Hodgkinson R.A., Barberis E., Presta M., Hartikainen H., Quinton J., Miller N., Sisák I., Strauss P., Mentler A. (2007). An environmental soil test to estimate the intrinsic risk of sediment and phosphorus mobilization from European soils. Soil Use Manage..

